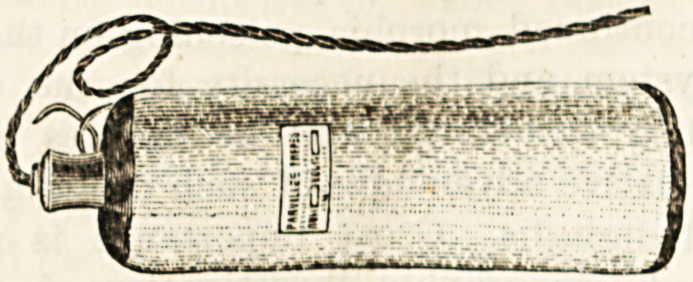# New Appliances and Things Medical

**Published:** 1901-12-21

**Authors:** 


### NEW APPLIANCES AND THINGS MEDICAL.

[We shall be glad to receive, at our Office, 28 & 29 Southampton Street, Strand, London, W.C., from the manufacturers, specimens of all new preparations and appliances which may be brought out from time to time.]

### A NEW TYPE OF ELECTRIC FOOT AND BED WARMER.

One of the latest uses to which electricity has been put is that of supplying heat to footstools and foot-warmers for the bed, and both of these adaptations are highly desirable in certain places and under certain conditions. The hot-water foot-warmers have the great disadvantage of not maintaining their temperature, and of requiring to be changed every few hours, thus involving the risk of being too hot at one time and not hot enough at another. These electric warmers have none of these drawbacks. Where an installation exists they are always ready; can be brought into use in a moment, and they maintain their temperature as long as the current is allowed to enter them. It is evident that for hospitals and the sick room generally this new application will be of the highest service to those concerned in nursing; and we hope that the inventors, Messrs. H. von Kramer and Co., of Bath, will not fix too high a price for these bed-warmers, or the hospitals, which ought to be their chief customers, will hesitate to buy what would be a most useful part of nursing armamentarium. The apparatus is practically indestructible, and the amount of electricity consumed is about the same as an ordinary 16-candle power light.

## Figures and Tables

**Figure f1:**
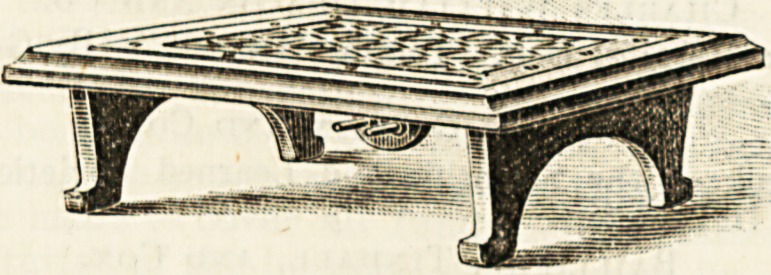


**Figure f2:**